# Are prehospital treatment or conveyance decisions affected by an ambulance crew’s ability to access a patient’s health information?

**DOI:** 10.1186/s12873-015-0054-1

**Published:** 2015-10-07

**Authors:** Ollie Zorab, Maria Robinson, Ruth Endacott

**Affiliations:** South Western Ambulance Service NHS Foundation Trust, Abbey Court, Eagle Way, Exeter, Devon, EX2 7HY UK; Centre for Health and Social Care Innovation, Faculty of Health, Education and Society, University of Plymouth, 8 Portland Villas, Drake Circus, Plymouth, PL4 8AA UK

**Keywords:** Ambulance, Paramedic, Clinical pathways, Health information, Medical records

## Abstract

**Background:**

A shift from a predominantly emergency service, towards one where a wide range of conditions are managed and treated on scene presents numerous challenges for ambulance services and clinicians. The effective management of a broad range of patients and conditions in the ambulance setting will have an impact on other parts of the health service including emergency departments and primary care.

**Methods:**

A two part online survey was distributed to operational staff working for a regional UK ambulance service. Clinicians were asked to report their experiences of accessing patient information and making decisions about patient management based on four hypothetical patient scenarios.

**Results:**

A survey of clinical staff (*n* = 302) revealed that (i) the vast majority experienced difficulties in accessing patients’ health information, (ii) this was particularly true in the out of hours period and (iii) They felt that better access would likely lead to more appropriate selection of care pathways.

**Conclusions:**

Decisions regarding the most appropriate care for patients presenting to the ambulance service are best informed by access to accurate and complete health information and records. An understanding of patients’ pre-existing medical conditions, recent treatments and health information is needed for the selection of the most appropriate care; this information is often difficult to obtain in the ambulance service setting.

**Electronic supplementary material:**

The online version of this article (doi:10.1186/s12873-015-0054-1) contains supplementary material, which is available to authorized users.

## Background

South Western Ambulance Service NHS Foundation Trust (SWASFT) is one of the largest regional ambulance services in England, serving a resident population of 5.3 million people spread over 51,000 km^2^. Traditionally, National Health Service (NHS) ambulance services were seen as providers of emergency care to those who are critically ill or seriously injured and as a means of transport to hospital. Increasingly, the service plays a crucial role in delivering care to those with urgent needs in relation to both acute and chronic medical presentations and also those with social and mental health care needs.

Providing the most appropriate care for these individuals can involve referral to, and liaison with, other healthcare professionals (HCP) and services. In some cases it can involve advice or the provision of care on scene without the need to convey the patient to hospital. Clearly, this shift from managing critically ill and seriously injured, towards a broader range of management options, means that clinicians are faced with a complex set of considerations when deciding on the best care pathways for many of the patients that they attend. Selection of the most appropriate care pathway is informed by assessment of the presenting patient, and by taking into account a host of other factors including past medical history, previous investigations and hospital attendances or end of life care wishes. Reports from staff suggesting difficulties with access to patient health information, prompted SWASFT to undertake a survey to discover more about how ambulance staff access health information, what information clinicians found most useful and whether or not improving access to information helps to ensure that patients are offered the most appropriate care pathway.

Transforming Urgent and Emergency Care Services in England [1] made several recommendations to try to improve urgent and emergency care. This included the recommendation that NHS 111 clinicians (who respond to urgent care phone calls) should have knowledge about patients and their medical problems, so the staff advising patients can provide relevant advice to help them make the best decisions. Although this recommendation was made for telephone based clinicians, the theory behind improving information access could be equally applicable for ambulance and emergency staff. In addition, the report highlighted the importance of this information for patients with long-term conditions or rare disorders, and those who are receiving end of life care.

The more recent ‘Ripping off the Sticking Plaster’ report [2] highlights the growing strain on Emergency Departments. The paper highlighted that access to urgent and emergency care can be complicated, and this can lead to delays in accessing the most appropriate care. The report promoted greater support for paramedics to help make decisions about when to convey patients to an emergency department, reducing inappropriate attendance and admission.

## Methods

The aims of the study were to:

1. Identify how ambulance clinicians currently access health information and barriers that prevent crews accessing data.
2. Ascertain whether a lack of information could lead to a suboptimal care pathway being selected.
3. Explore whether, in hypothetical scenarios, increasing the amount of information available would lead to selection of a more appropriate care pathway.

### Survey Instrument

Following an extensive review of the literature, an online, two-part questionnaire was created (please see copy at Additional File 1). Respondents were asked to report their experiences of accessing patient information (Part 1) and make decisions about patient management based on four hypothetical patient scenarios (Part 2). The scenarios were designed to examine whether the absence of patient information would be likely to alter their clinical decisions. This scenario-based approach is commonly used to gain insight into clinical decision-making [3]. (The four scenarios are at Table 6).

Some responses required the respondent to provide a rating using a Likert scale. Free text boxes were available at the end of each section for participants to provide (optional) additional comments. Due to the nature of ambulance deployment, crews often have limited discretionary time on an ambulance station, for this reason the survey was designed to be completed in a short space of time using a selection of multiple choice and drop down answers.

### Data collection and statistical methods

A link to the online questionnaire was distributed to operational staff across all sectors of the Trust; this covers Cornwall, Devon, Somerset, Dorset, Wiltshire, Gloucestershire and the former Avon area. A link to the survey was initially emailed to every front line member of staff within the Trust, this included Emergency Care Assistants, Ambulance Technicians, Student Paramedics, Paramedics, Emergency Care Practitioners and Critical Care Paramedics. A further two reminders were published within the Trust’s weekly Chief Executive’s Bulletin, which is sent electronically to all staff.

### Statistical methods

Data was analysed using the Statistical Package for the Social Sciences (SPSS) version 21. Descriptive statistics were used to summarise findings, with Chi square used to examine associations between demographic data items and responses.

### Ethics

The survey was reviewed by the Trust Research and Development Group and approval was obtained. NHS Research Ethics Committee approval was not required as the survey was completed by staff only [4]. Written information was provided with the link to the electronic survey; staff were deemed to have consented to participate if they chose to complete the survey.

## Results

The online survey was available to be completed for 31 days; during this time 302 clinicians completed the demographic data section and Part 1 of the survey and 285 completed the entire survey. Given that the survey was completed at the ambulance station, some attrition was expected. Allowing for some staff absence during the data collection period (annual leave, study leave, sick leave), it is estimated that the potential pool of respondents was 2700. This represents a response rate of 12.0 %.

Of the 302 respondents, 63 % (176) were male and the majority entered their current job role via the Institute of Health Care Development (IHCD) rather than Higher Education Institution (HEI) route (65.5 % : 34.5 %). Most respondents were aged 26–55 (85.6 %) with 41.5 % (*n* = 118) aged 35–45 years. Clinical grades were grouped into Advanced Practitioner (emergency care practitioner or critical care paramedic) (36/12.9 %), Registered Practitioner (paramedic, clinical support officer or clinical team leader) (185/66.3 %) and Unregistered Practitioner (emergency care assistant, advanced technician, ambulance practitioner or student paramedic) (58/20.8 %). Clinical grades and length of service were comparable in respondents and in the overall population. Length of service for the population and length of service/duration of current role for respondents is at Table 1.

**Table 1:** Length of time in ambulance service and current role

	Years (% of respondents)				Years (% of population)			
	0–2	3–6	7–15	16+	0–2	3–6	7–15	16+
Length of time in the ambulance service	14 (5.0)	58 (20.6)	134 (47.5)	76 (27.0)	69 (3.4)	396 (19.7)	909 (45.3)	629 (31.4)
Length of time in your current role	66 (23.2)	93 (32.6)	93 (32.6)	33 (11.6)				

### Part 1

Overall, 285 (94.4 %) of respondents felt that they had been unable to access health information about a patient that they were caring for, whilst 274 (90.7 %) felt that this lack of information had led to a less appropriate care pathway being selected and the majority (246/81.5 %) felt that information was easier to access during working hours on weekdays. When asked what type of information was not available, which could have helped them make a decision (see Table 2), 262 (86.8 %) of respondents recalled that a patient’s past medical history was not readily available whilst 233 (77.2 %) were unable to gain timely access to a patient’s resuscitation status or end of life care wishes. Responses categorised as ‘other’ were predominantly most recent ECG (*n* = 15), previous summary from General Practitioner (GP)/hospital other HCP (*n* = 10) or mental health information (*n* = 8).

**Table 2:** Information that respondents perceived might have helped the decision making process

Information category	*n* (%)
Resuscitation status	233 (77.2)
Current medication	184 (60.9)
Allergy information	103 (34.1)
Previous medical history	262 (86.8)
Patient’s normal parameters	235 (77.8)
End of life care choices	221 (73.2)
Information about implanted	
Devices e.g. pacemakers	106 (35.1)
Other^a^	38 (13)

^a^Other sources of information identified by respondents included: ECGs, mental health records, blood and other test results, family history, next of kin details, recent medical or hospital attendances.

Sources that respondents use when trying to access a patient’s health information were mainly the patient’s GP (*n* = 294, 94.7 %) whilst only 157 (52 %) indicated that they would consider contacting an out of hours urgent care provider for information about a patient (see Table 3).

**Table 3:** Sources used when trying to access patient health information

Sources used when trying to access health information	*N* [%]
GP	294 [97.4]
Out of hours provider	157 [52.0]
Clinical hub	107 [35.4]
Message in a bottle scheme^a^	196 [64.9]
Other	54 [17.8]

^a^A regional initiative - the bottle is part of an information pack and contains a form for storing medical information, contact details and a description of existing medical conditions

Participants were then presented with examples of information sources that ambulance clinicians could use to find out more about a patient’s health. This included a recent hospital discharge summary, GP summary, GP last consultation notes, district nursing notes and a child health record. Respondents were then asked to rate each piece of information using a Likert scale using the terms Most Helpful, Helpful, Neither Helpful or Unhelpful and finally, Unhelpful. Hospital Discharge and GP Summaries were the two pieces of information which score highest with 90 % describing them as either ‘Most Helpful’ or ‘Helpful’ (see Table 4).

**Table 4:** Information sources rated in terms of perceived helpfulness

Information sources rated in terms of helpfulness	Most helpful/helpful *n* [%]	Neither helpful or unhelpful *n* [%]	Unhelpful *n* [%]
Hospital discharge summary	269 [90.9]	21 [7.1]	6 [2]
G.P. summary	266 [89.8]	28 [9.5]	2 [0.7]
G.P. last consultation notes	259 [87.5]	35 [11.8]	2 [0.7]
District nursing notes	217 [73.3]	75 [25.3]	4 [1.4]
Child health record (red book)	208 [70.3]	81 [27.4]	7 [2.4]
Other* *n* = 52			

*Other useful sources identified by participants included: patient report, family members, carers, medications and letters visible to staff.

### Part 2

The second part of the survey presented participants with 4 hypothetical scenarios to try to understand whether or not increasing the amount of information available to clinicians might lead to a more appropriate care pathway being selected. Each question had a brief introduction followed by an initial management question where respondents were asked to answer using a 5 point Likert scale, ranging from Very Unlikely to Very Likely. Further patient health information was revealed, and the participants were then asked for another management decision, this answer was again provided using a 5 point Likert scale (see Table 5).

**Table 5:** Hypothetical scenarios used in the survey

Hypothetical scenario	Initial management question	Additional management question
1. Following a clinical examination of a patient, you feel that their condition could be safely managed in the community.	a. How likely would you be to try to obtain details of allergies and a current medication list before contacting and referring to an alternative care provider?	a. You are unable to find details of allergies and what medication the patient is taking. How likely is it that you would still continue with the referral to the alternative care provider?
2. You respond to a patient who was seen acting strangely before having a convulsion. On arrival the patient is unresponsive. You find a wristband containing information stating that the patient is a resident at a local supported living project and has epilepsy. Previously after a seizure, they have made a full recovery within 30 minutes.	a. How likely is it that you will consider this information before making a decision whether or not to convey to the emergency department?	c. How likely is it that you would have made the same decision without the additional information provided by the wristband?
	b. How likely is it that you would remain on scene for up to 30 minutes before making a decision whether or not to convey this patient to hospital?	
3. You respond to a patient who has fallen and is short of breath with a productive cough. The patient has information at home which states that they have COPD and gives details of observations taken when they were last discharged from hospital. This information includes SpO2 levels, which is similar to the observations you have just recorded.	a. How likely is it that you will consider this additional information when deciding whether or not to convey this patient to hospital?	a. How likely is it that you would have made a similar conveyance decision without the additional information provided by the discharge summary?

*Treatment Escalation Plan (TEP) - regional initiative linked to patients’ resuscitation wishes.

Subsequent to the examination of the distributions of the responses, categories were grouped to allow easier visualisation of the data (Table 6). Inferential tests revealed relationships between demographic data and usefulness of data. Training route was significantly associated with: the usefulness of the GP summary (chi _2_ 14.246, *p* =0.027), notes from the last GP consultation (chi _2_ 17.100, *p* =0.009) and District Nursing notes (chi _2_ 12.628, *p* =0.049). In each instance, respondents who entered their current job via higher education were more likely to use these additional sources of information.

**Table 6:** Responses (%) by hypothetical scenarios

Scenario/question	Very unlikely/unlikely	Possibly	Likely/very likely
1. Patient appropriate for community care			
a) Try to obtain allergies/medications information before referral?	24 [7.3]	56 [18.8]	216 [72.9]
b) Unable to access allergies/ medications information – proceed with referral?	42 [14.2]	147 [49.7]	107 [36.1]
2. Patient with convulsion			
a) Consider information on patient bracelet?	5 [1.7]	30 [10.3]	255 [88.0]
b) Use info to remain on scene for 30 minutes?	27 [9.3]	86 [29.7]	177 [61.0]
c) Same decision without bracelet information?	193 [66.6]	70 [24.1]	27 [9.3]
3. Patient with chronic obstructive pulmonary disease (COPD) and fall			
a) Consider information re. normal observations to inform conveyance decision?	3 [1.0]	25 [8.7]	261 [90.3]
b) Same decision without additional information from discharge summary?	121 [41.9]	127 [43.9]	41 [14.2]
4. Terminal patient with documented end of life care (EOLC) wishes stating that they do not wish to be resuscitated			
a) Convey patient to hospital despite documented wishes?	224 [77.8]	53 [18.4]	11 [3.8]
b) Same conveyance decision if EOLC document not in place?	171 [59.3]	66 [22.9]	51 [17.8]

There were some associations between demographic data and responses to the scenario questions. Referral decision taken without information regarding allergies and medications was significantly associated with age (chi _2_ 24.221, *p* =0.007). Usefulness of information about the patient’s normal vital signs (for a patient with COPD) was associated with length of time in the ambulance service (chi _2_ 41.434, *p* = <0.001) and clinician grade. Use of Treatment Escalation Plan information to inform conveyance decision was associated with length of time in the ambulance service (chi _2_ 19.272, *p* =0.013).

## Discussion

It is widely recognised in emergency medicine that access to current patient health information is critical for safe practice [5]. Studies looking at how emergency clinicians access information have concluded that improved access to health information can improve patient safety and can save a significant amount of time making consultations more efficient [5–7]. The evidence provided by this survey suggests that ambulance clinicians could also benefit from improved access to patient information. The survey revealed that crews felt that they are not always able to access the patient information required to make appropriate conveyance decisions. When questioned, information such as end of life care wishes, resuscitation status and previous medical history are often required by ambulance staff to ensure that the most appropriate care pathway is offered. The survey has also suggested that a lack of this information can result in a less appropriate care pathway being selected.

Findings revealed that a patient’s previous medical history, normal parameters and resuscitation status were the most common pieces of information that ambulance clinicians felt that they were unable to access in a timely manner. Most of this information is held by a patient’s doctor and can be readily obtained by ambulance clinicians by contacting a GP for advice. This professional relationship between ambulance crews and local GPs is further demonstrated later on in the survey when nearly all respondents state that they currently utilise a patient’s GP when trying to access patient information. This relationship between emergency clinicians and primary care physicians is known to be beneficial, especially for the management of frequent service users where improved information sharing can enhance care and organisational efficiency [8]

During the out of hours period (evenings and weekends), contacting a patient’s usual GP is often not an option and urgent primary care is provided by locally contracted out of hours providers [9]. It is during this time that ambulance clinicians encountered more difficulties accessing patient information. Traditionally, urgent care providers such as out of hours GP services and national NHS helplines do not have access to a patient’s records so will be unable to provide the same information to that of a patient’s registered GP [6]. This lack of available information or previous negative experience could account for why a smaller number of clinicians indicated that they utilised an out of hours urgent care provider to find out more about a patient’s health information compared to contacting an in-hours GP when available.

The lack of timely access to a patient’s health information in the out of hours period is not a problem that will be unique to front line ambulance staff [6]. Following the introduction of NHS 111, patients with urgent primary care needs now have to contact 111 to arrange to be seen by an urgent care provider [10]. NHS England [1] has already acknowledged the need for clinicians working for NHS 111 to have access to more patient specific information to help them decide on the most appropriate care option. However, as a result of the introduction of NHS 111, ambulance services have seen a rise in emergency ambulance activity [11] with crews often responding to patients with primary care needs or those who need help managing a long term condition. With ambulance clinicians now seeing more patients requiring primary care in the out of hours setting, this survey has demonstrated the growing need to ensure that crews have access to the information needed to help them select optimum, safe care pathways.

Like most ambulance services, SWASFT employs clinicians based within the control room (Clinical Hub) who can access a limited amount of patient information that has been shared by other health care organisations involved in a patient’s care; this can often include end of life care wishes and ‘do not attempt resuscitation’ instructions. Although a large number of clinicians indicated that they require access to a patient’s end of life care wishes and resuscitation status, few indicated that control room practitioners were utilised as an information source. Although control room staff are relatively easy to access and available around the clock, the information held is reliant on external organisations to keep it up to date and relevant. Nevertheless, this disparity highlights a potential area for development allowing centrally based ambulance clinicians access to up to date patient information which could support practitioners delivering care at the patient’s side.

The lack of systems for data sharing between healthcare agencies has been the subject of much criticism both in the NHS [12] and overseas [13]. Whilst achievements in the GP sector of primary care are notable [14], this does not include the ambulance service. One solution might be to expand the patient-held records system used in maternity services for the primary care patient population. The research agenda to accompany such an initiative might include feasibility, acceptability and usefulness for clinicians, patients and carers; the relative risks and merits of paper and electronic approaches; and the relative cost benefits.

As the paramedic profession has developed, initial education and training has shifted from traditional ‘in-house’ courses accredited by the IHCD to University based degrees. It is hoped that by raising the standard of paramedic education, it is possible to provide clinicians with greater underpinning knowledge, competencies and clinical practice experience to provide appropriate treatment, referral or discharge plans for patients [15]. As part of the survey, demographic data was collected which suggested a significant relationship between a paramedic’s training and how useful they found pieces of information such as a GP summary or District Nursing notes. Paramedics who had completed higher education training found additional sources of information more useful compared to clinicians who had entered following an IHCD course. This interesting finding could be attributed to a number of factors, including improved education and understanding of alternative care options or a graduate paramedic’s initial lack of experience resulting in reliance on external information sources.

During the second part of the survey, hypothetical scenarios were introduced which attempted to ascertain whether or not the provision of additional information would lead to the selection of more appropriate care pathways. The scenarios were broadly themed to cover community care, long term conditions, and end of life care wishes. In nearly all of the scenarios, the responses suggest that management, and in particular conveyance, decisions were altered by providing clinicians with additional information. This can be most clearly seen in the final scenario which presents a patient in the final stages of an illness. The responses from clinicians suggest that without the addition of documented end of life care wishes a patient in the final stages of an illness may still be conveyed to an emergency department. This particular scenario will be familiar with most prehospital staff who may have previously encountered patients in the final hours of their life. Without documentation to support a decision, ambulance clinicians will often convey patients to an emergency department which could cause further distress to relatives. Access to a patient’s resuscitation status and end of life care wishes was frequently mentioned during the survey as a piece of information that was not readily available when required.

### Limitations

This survey only looked at one regional ambulance service and may not be representative of practice experienced in other parts of the UK. In addition, the information gained was based on individual paramedic’s perceptions and experiences of accessing information and is therefore subjective in nature.

The low response rate of 12 % could also be viewed as a limiting factor. When the survey was distributed, the ambulance service was experiencing a period of high operational demand. This reduced the amount of time crews spent on station, affecting their availability to complete the survey. Following the initial email inviting staff to take part, additional reminders were placed in the Trust’s Bulletin published weekly. However, no further reminder emails were sent which could have contributed in the reduced response rate.

## Conclusions

This survey provides additional evidence to support the view that ambulance clinicians are not always able to access health information regarding patients that they are caring for. Accurate health information is vital to make safe conveyance decisions and a lack of access could result in patients being unnecessarily conveyed to an emergency department when alternative care pathways may be appropriate and available.

Evidence from hospital based practice has demonstrated that decisions regarding the most appropriate care for patients presenting with urgent primary care needs are best informed by access to up to date health information and records. Without this direct access, paramedics and other ambulance staff have developed professional relationships with other health care professionals to enable them to find out more about the patients that they are caring for. This will often involve a patient’s GP or community based staff not available in the out of hours setting.

An understanding of patients’ pre-existing medical conditions, recent treatments and health information is needed for the selection of the most appropriate care; this information is often difficult to obtain in the out of hospital setting, and in particular, outside of usual office hours. Evidence from hypothetical scenarios used in this survey suggests that without this information suboptimal care pathways could be selected placing even greater pressure on hospital emergency departments.

To enable paramedics to safely manage more patients within the community, ambulance services and other care providers need to work together to develop mechanisms that allow clinicians to access patient health information whilst at the patient’s side. This could be achieved by making use of existing out of hours urgent care providers or NHS 111. In addition, clinicians embedded within the ambulance control room could be further utilised to provide support to field based staff to provide them with the information required to select the optimum pathway every time. In the medium term, opportunities to develop patient-held records should be explored.

## Additional file

Additional file 1:
**Accessing Information Survey.** (DOCX 25 kb)

## Abbreviations

DH: Department of Health
ECG: Electrocardiogram
GP: General Practitioner
HCP: Health Care Professional
HEI: Higher Education Institution
IHCD: Institute of Health Care Development
NHS: National Health Service
SPSS: Statistical Package for the Social Sciences
SWASFT: South Western Ambulance Service NHS Foundation Trust
UK: United Kingdom
