# Supplementary material for: Are prehospital treatment or conveyance decisions affected by an ambulance crew’s ability to access a patient’s health information?
Source: BMC Emerg Med. 2015 Oct 7;15:26. doi: 10.1186/s12873-015-0054-1 (PMC4596371; doi:10.1186/s12873-015-0054-1)
Supplement: Additional file 1: — Accessing Information Survey. (DOCX 25 kb) [file 12873_2015_54_MOESM1_ESM.docx]

**Accessing Information Survey**

**Introduction**

South Western Ambulance Service NHS Foundation Trust is seeking to improve the way ambulance clinicians are able to access information about the patients that they care for.

As part of this work, and to inform a larger research project, we are conducting a survey to help us understand some of the barriers that currently exist when ambulance staff are trying to access patient information.

This survey will present you with various fictitious hypothetical scenarios to try and establish whether or not gaps in the information available to clinicians lead to a different outcome or care pathway being selected.

The survey is anonymous; we ask for the operations sector that you work in order to indentify local trends. This information will not be used to identify individuals participating in the survey.

Thank you for your time.

**Demographics**

Age

*18-25*

*26-34*

*35-45*

*46-55*

*56+*

Gender

*Male*

*Female*

Clinical Grade

*Emergency Care Assistant*

*Student Paramedic*

*Advanced Technician or Ambulance Practitioner*

*Paramedic*

*Clinical Support Officer or Clinical Team Leader*

*Emergency Care Practitioner*

*Critical Care Paramedic*

By which route did you join your current job role?

*IHCD*

*Higher Education*

Length of time in the ambulance service

*0-2yrs*

*3-6yrs*

*7-15yrs*

*16yrs+*

Length of time in your current job role

*0-2yrs*

*3-6yrs*

*7-15yrs*

*16yrs+*

**Accessing Information**

Have you ever felt unable to access information which you felt you needed to make a decision about the ongoing management of a patient?

*Yes or No*

Have you ever felt that an inappropriate care pathway has been selected due to you not being able to access information about a patient you are caring for?

*Yes or No*

Please select the statement below which best matches your experience of accessing patient information

*I find it easier to access patient information during working hours on weekdays.*

*I find it easier to access patient information during evenings and weekends*

*I find that access to patient information is not altered by the time of day or day of the week.*

If you have experienced cases when timely information wasn’t available, which information might have helped in your decision making process?

Please select from one or more of the following options:

*Resuscitation Status,*

*Current Medication,*

*Allergy Information,*

*Past Medical History,*

*Patient’s Normal Parameters,*

*End of Life Care Choices,*

*Information about implanted devices e.g. pacemakers*

*Other (please specify)*

What sources do you currently use when trying to access patient information?

*GP*

*Out of Hours Provider*

*Clinical Hub*

*Message in a bottle scheme*

*Other (please specify)*

The following are examples of information sources that ambulance clinicians can use to gain additional information about a patient's medical history and on-going

management plan. Please rate the examples below by selecting one of the following options Most Helpful, Helpful, Neither Helpful or Unhelpful, Unhelpful

*Hospital Discharge Summary*

*G.P. Summary*

*G.P. Last Consultation Notes*

*District Nursing Notes*

*Child Health Record*

*Other (please specify)*

**Part 2 - Hypothetical Scenarios**

Please answer the following questions using by selecting from the following options:

*Very Unlikely*

*Unlikely*

*Possibly*

*Likely*

*Very Likely*

Following a clinical examination of a patient, you feel that their condition could be safely managed in the community.

How likely would you be to try to obtain details of allergies and a current medication list before contacting and referring to an alternative care provider?

You are unable to find details of allergies and what medication the patient is taking. How likely is it that you would still continue with the referral to the alternative care provider?

You respond to a patient who was seen acting strangely before having a convulsion. On arrival the patient is unresponsive. You find a wristband containing information stating that the patient is a resident at a local supported living project and has epilepsy. Previously after a seizure, they have made a full recovery within 30 minutes

How likely is it that you will consider this information before making a decision whether or not to convey to the emergency department?

How likely is it that you would remain on scene for up to 30 minutes before making a decision whether or not to convey this patient to hospital?

How likely is it that you would have made the same decision without the additional information provided by the wristband?

You respond to a patient who has fallen and is short of breath with a productive cough. The patient has information at home which states that they have COPD and gives details of observations taken when they were last discharged from hospital. This information includes SpO2 levels which is similar to the observations you have just recorded.

How likely is it that you will consider this additional information when deciding whether or not to convey this patient to hospital?

How likely is it that you would have made a similar conveyance decision without the additional information provided by the discharge summary?

You are called to a patient who has terminal cancer, the patient’s family is

concerned that they may have a chest infection as their breathing has become laboured and they are unresponsive. You are presented with a 'Treatment Escalation Plan' which documents that the patient does not wish to be resuscitated, would prefer to die at home and does not wish to receive invasive ventilation or IV antibiotics. How likely is it that you will convey this patient to hospital?

How likely is it that you would have made a similar conveyance decision without the additional information in the Treatment Escalation Plan?

Do you have any further comments that you would like to make regarding how you access patient information?

Thank you for taking the time to complete this survey.
